# Meeting Report: Summit Focuses on Pharmaceuticals in Drinking Water

**DOI:** 10.1289/ehp.117-a16

**Published:** 2009-01

**Authors:** Tanya Tillett

Keeping our drinking water clean and contaminant-free is a concern of many policy makers and scientists, and it was the headlining topic at the inaugural Environmental Health Summit of the Research Triangle Environmental Health Collaborative held 10–11 November 2008 in Research Triangle Park, North Carolina. With nearly 150 attendees representing government, research, academia, public interest groups, and the pharmaceutical industry, the stage was set for a constructive dialogue designed to identify key issues for future discourse and action.

In his opening remarks to the conference attendees, chairman and former NIEHS director Kenneth Olden discussed the origins of the collaborative, originally conceptualized in 2005 as an independent forum that would advise government and private industry on emerging environmental threats and concerns. This goal helped shape the current summit’s focus on pharmaceuticals in drinking water, he said.

North Carolina congressmen Bob Etheridge and David Price also spoke, as did experts from the U.S. Geological Survey, Food and Drug Administration, and Environmental Protection Agency (EPA), who provided scientific background and information on the current regulatory landscape regarding pharmaceuticals in drinking water supplies. Only 1 pharmaceutical—nitroglycerin—appears on the EPA’s current Drinking Water Contaminant Candidate List. John Sumpter, head of the Brunel University Institute for the Environment in the United Kingdom, discussed the European perspective on the issue, where there is still limited regulation but greater awareness of the issue, with media covering the problem more extensively than U.S. outlets.

An estimated 41 million Americans are exposed to trace pharmaceuticals in their drinking water, according to the results of an Associated Press investigation published online 9 March 2008. A significant amount of these trace pharmaceuticals are unmetabolized drugs that are excreted as waste. Others are unused medications that people have flushed down the toilet. Veterinary drugs, usually from large farming operations, are also present in drinking water. Not all water treatment methods currently in use can remove all pharmaceuticals in water.

Jim Hagan, vice president of GlaxoSmithKline’s Environment, Health and Safety Department, says the pharmaceutical industry has known about this problem for years. “[O]ur internal working groups routinely assess both the environmental fate and effects [of new products]. The only difference is that now the analytical sciences have advanced to the point where we can accurately measure parts per trillion in water.”

Rachel Golden-Smith, an education program manager with the North Carolina Department of Environment and Natural Resources, pointed out the advantages of direct communication between pharmacists and consumers to determine how different medications should be discarded. When people are given enough digestible information about the hazards of flushing medications down the toilet, they are less likely to do so, she says. She adds, however, that “drug return programs may incorrectly imply to the public that by returning their unused drugs, the problem of pharmaceuticals in the water is being solved. It is important to balance providing avenues for people to help solve the problem with honest communication about where most pharmaceuticals in the water are coming from.”

The attendees participated in 4 work-group sessions throughout the 2-day summit to articulate a plan of action for future areas of focus. On the second day, the group made and prioritized recommendations based on their discussions. The highest priorities included starting a public policy discussion of the issue and drafting a brief written response that water treatment facilities and pharmacies can provide to consumers who ask about the safety of their drinking water: “If your drinking water meets current U.S. standards, your drinking water is considered safe and drinkable. However, trace amounts of pharmaceuticals and other chemicals have been found in water. These substances are coming from a variety of sources and are difficult to remove. There is limited information on how they affect humans and wildlife. U.S. standards for safe drinking water may need to change as more information becomes available.” The collaborative is also developing a website with more information on pharmaceuticals in drinking water.

Olden said the collaborative also plans to brief Congress on the outcome and goals of the summit and will submit the conference proceedings to a peer-reviewed journal for publication. For more information on the collaborative and the summit, visit http://www.environmentalhealthcollaborative.org/.

## Figures and Tables

**Figure:**
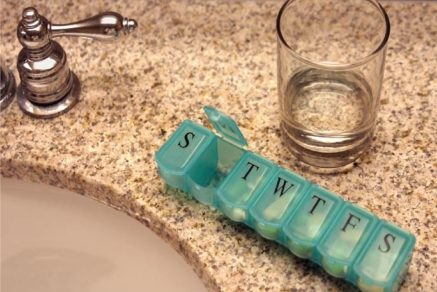
Traces of pharmaceuticals end up in drinking water by a variety of routes.

